# The waste ban in China: what happened next? Assessing the impact of new policies on the waste management sector in China

**DOI:** 10.1007/s10653-021-01101-y

**Published:** 2021-11-18

**Authors:** Na Song, Iain McLellan, Wei Liu, Zhenghua Wang, Andrew Hursthouse

**Affiliations:** 1 School of Computing, Engineering and Physical Sciences, University of the West of Scotland, Paisley, PA1 2BE UK; 2 Waste Disposal Supervisory Office of Zhuzhou, Zhuzhou, 412000 China; 3 Hunan Provincial Key Laboratory of Shale Gas Resource Utilization, Hunan University of Science and Technology, Xiangtan, 411201 China

**Keywords:** Solid waste, Waste regulation, Waste import, Small business, COVID19 impact on waste

## Abstract

The 2017 ban on the waste import and new policies for the waste management sector in mainland China had wide-spread impact. After decades of poor environmental and public health impacts from the sector, a study is needed which focuses on policies updates and waste management. This provides a direction for the survival of local waste management industries and consider similarities with the ban promulgated in China on the restriction of waste import from other countries. We review the waste management situation in China before national legislation prevented the import of waste, highlight the status of landfill mining in China, and review the dynamics of domestic policies before and after the promulgation of the ban in China. The impact of the COVID19 pandemic on the waste management system is starting to emerge, providing both challenges and opportunities for the sector in China. We see the impact of the ban on the range of imported waste and domestically generated materials. The ban results in price increases for domestic recycling that forces companies to introduce more formal recycling processes and to drive the consumption behaviours to more reasonable and environmentally friendly options. The driver in China is to reduce pollution in the environment and improve health, but a negative impact has been from increased landfill mining which has impeded the original aim of the waste ban and requires further technological development. The dynamic of domestic policies in China shows higher level of activity of updates and revisions or introduction of new policies from 2015 onwards and the concept of ‘zero waste cities’ brings new hope for improvement of the Chinese waste management system. The pandemic also suggests an important step to establish sustainable management systems despite evidence of increased “fly-tipping”. The rebound of the waste ban may have stimulated in the short term negative impacts on local environments both in China and internationally.

## Introduction

Waste management is still a challenging global issue because of a rapid increase in the amount of waste produced as a result of rapid economic growth, development of technology, population growth, and overconsumption. Around 2.01 billion tonnes of waste was generated worldwide in 2016, and the waste generation is expected to increase to 3.40 billion tonnes annually by 2050 (Kaza et al., 2018). Solid waste is typically recycled in developed countries, exported to developing countries, and has provided a source of income as a resource. However, a ban on the import of foreign waste and reform of the administrative system for solid waste was announced by the Chinese government in 2017. This raises a number of questions: What has the impact of the solid waste ban in China been on the internal waste management industry? Has the ban achieved its target of environmental protection and improvement of human health? Will the approval and acceptance of applications for the import of solid waste finally be stopped from 2021 (Creech, 2020)?

China’s solid waste ban not only changed the global recycled solid waste supply chain, which diverted solid waste to other markets, like Malaysia and other parts of Southeast Asia (Tran et al, 2021); but also led to the construction of new infrastructure for solid waste management in these new destination countries to provide the capacity to handle sudden growth in waste streams (Hook & Reed, 2018). In addition, the diverted plastic scrap appears to be handled by small-scale waste processors, operating under little to no environmental regulations (Hook & Reed, 2018). It also increases the flow of potentially hazardous waste, for example, Waste Electrical and Electronic Equipment (WEEE) into Thailand that recycled the waste into new plastic materials (Hook & Reed, 2018). As a result, Malaysia, Vietnam, and Thailand are due to freeze the import of solid waste, which will lead to major waste producers such as the USA, UK, Japan, and Australia facing the challenge to establish alternative mechanisms to deal with the solid waste internally (WMR, 2018).

The introduction of the ban has locally initiated a positive impact on the environment in China and, with an increased demand for long-term sustainable development (United Nations, 2015), has resulted in waste management companies switching their target material or stopping their operations in the short term (Qu et al., 2019). However, there are challenges for the survival of companies that use solid waste plastic as resource material in China: a lot of small groups and some waste management companies relying on imported waste as a feedstock have had to stop trading due to a shortage of raw material (Schulz, 2020) while some waste management companies moved to other countries to continue their business after the ban (Parajuly & Fitzpatrick, 2020). Immediate and direct impacts of the Chinese import ban have highlighted that an assessment for evaluating shipment policies linked to waste management is still required for a more comprehensive long-term impacts rather than the short-term economic benefits (Parajuly & Fitzpatrick, 2020).

**Fig. 1:**
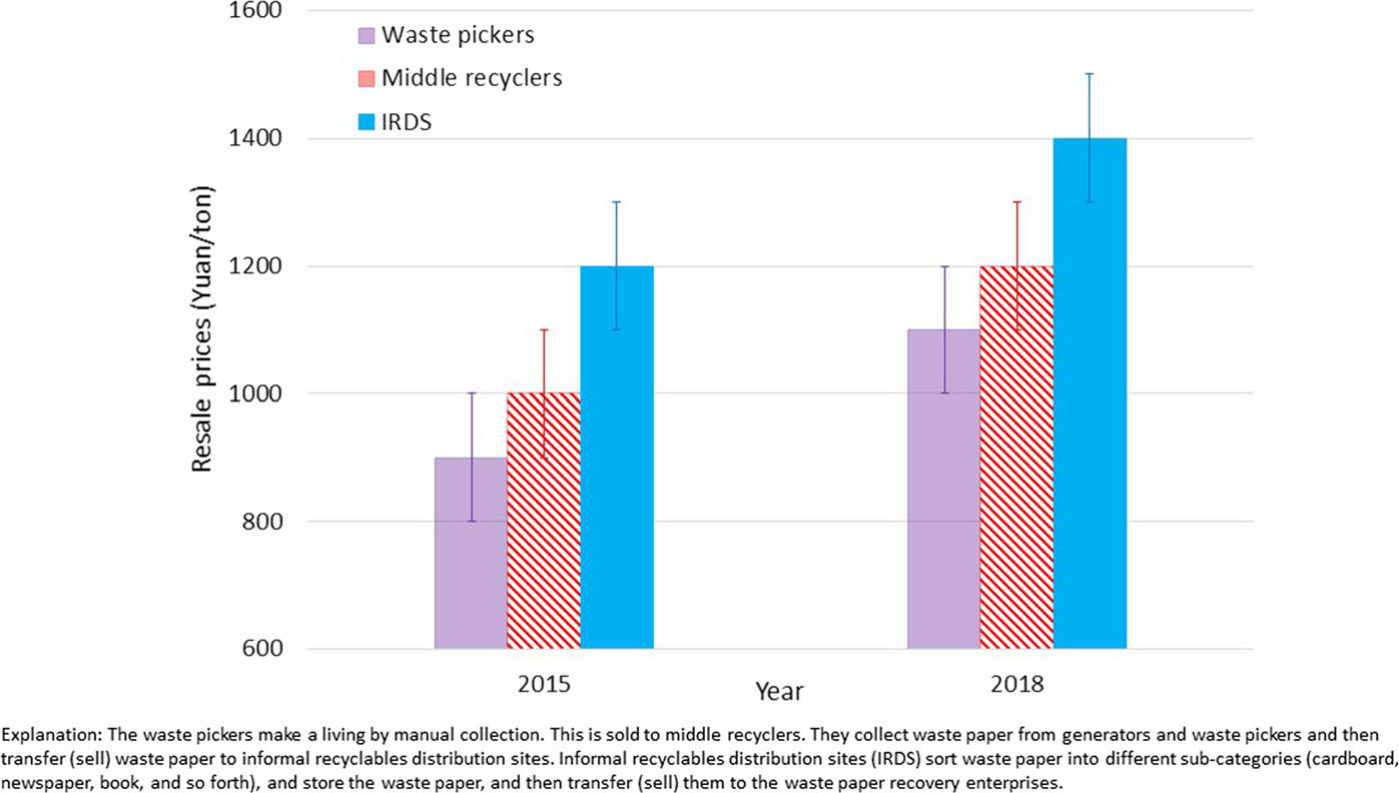
Comparison of resale prices (2015 and 2018) for waste paper for stakeholders in the paper recovery supply chain in Beijing, China (Yang et al, 2020)

As a case study, the sustainability of waste paper recycling was analysed for Beijing city (Yang et al., 2020), and the results indicated that informal recycling accounted for nearly 80% of waste paper recycling. This showed an increase in the price of waste paper resale from waste pickers to middle recyclers and onward to informal recyclate distribution sites (IRDS) and finally to paper recovery enterprises. In Fig. 1, the data is presented for 2015 and 2018 to provide clear indicators of the market shift. In addition, the average distance from the city centre (waste source) to distribution sites for informal recyclate has increased from 27.5 to 40.9 km, and the number of these sites has decreased from 27 to 11. Each stakeholder in the supply chain still receives a net profit, which means that the value chain for wastepaper recycling can be regarded as sustainable (Yang et al., 2020). A nontrivial challenge would come if the recycling cost keeps growing in the future according to the scenario analysis (Yang et al., 2020).

There has been a significant gap in the strength of environmental policies and regulations between a developed country and developing countries like China (as per United Nations definitions) which makes waste trade possible based on the difference in regulations and financial costs in each country. The evolving Chinese waste management system is separated as municipal solid waste (it is also called domestic garbage) management, industrial solid waste, and hazardous waste. There has been a rapid increase in Chinese domestic waste production over the last two decades with the increase of municipal solid waste (MSW) from 214 million tons (135 million tons in Chinese cities and 79 million tons in counties) in 2001 to 311 million tons (242 million tons in Chinese cities and 69 million tons in countries) in 2019 (MHURD, 2020). The impact of the ban on domestic Chinese and global waste flows has been analyzed (Huang et al., 2020) using (i) An structural path analysis combined with a multi-regional input–output model to know how the consumption patterns drive plastic waste import to China, (ii) A ecological network analysis to identify dominant controller in the global plastic waste trade network, (iii) A hypothetical extraction method to understand the added value for China and increased waste treatment capacity requirement for other economies. The results indicated that there was a lack of recycled plastic material due to the import ban, which accounted for 28.1% of domestic plastic waste recycling. The key controllers of plastic waste flow are China, the US, Germany, and the wider EU, and it is difficult for other large economies to replace China’s role within the trade network in the short term. This has a higher relative impact on the developed countries, which need to rapidly increase their waste treatment capacity than for the situation in China (Huang et al., 2020).

Environmental impacts from mechanical recycling of waste plastics, incineration, and landfill with municipal solid waste were evaluated with a life-cycle assessment (Chen et al., 2019). This demonstrated the environmental benefit of the current treatment in end-of-life (EOL) waste plastics through the analysis of current recycling technologies and the impact of the ban. It was found that the ban decreased the transport distances of waste plastics, which thereby reduces related environmental impacts such as a reduction of 84.8% marine eco-toxicity potential (Chen et al., 2019). A challenge is that more than 95% of the labelled plastic is associated with WEEE, which creates additional hazards when mechanical recycling is used (Hook & Reed, 2018). The wider environmental impact is influenced by a number of important factors: (i) the percentage of the recyclable plastic within the imported waste, (ii) whether it is recycled or goes straight to landfill, and (iii) illegal import (smuggling) of WEEE in containers falsely documented as containing plastic waste (Hook & Reed, 2018).

It is quite reasonable to assume that China announced the ban to protect the domestic environment and improve human health, and a number of studies have been carried out to assess the impact. However, the positive effect is negated because of a number of internal concerns. Growing Chinese urbanisation produces a lot of constructions and demolition waste which is sent to landfills, therefore there is an increasing need for landfill space. This waste is also secretly, and illegally, dumped in the countryside (“fly-tipping”), becoming more common in many jurisdictions during the covid-19 pandemic, with a direct impact on the environment and citizens’ health. Data on this is relatively poorly developed in the region (Lee et al, 2021). However, to reduce this environmental impact, new regulations are necessary to force further processing with only residual waste to be landfilled. We provide a perspective on issues impairing the positive impact of the waste ban in a domestic context, considering the landfill mining situation in China, urbanisation, and policy change.

## Materials and methodology

**Fig. 2:**
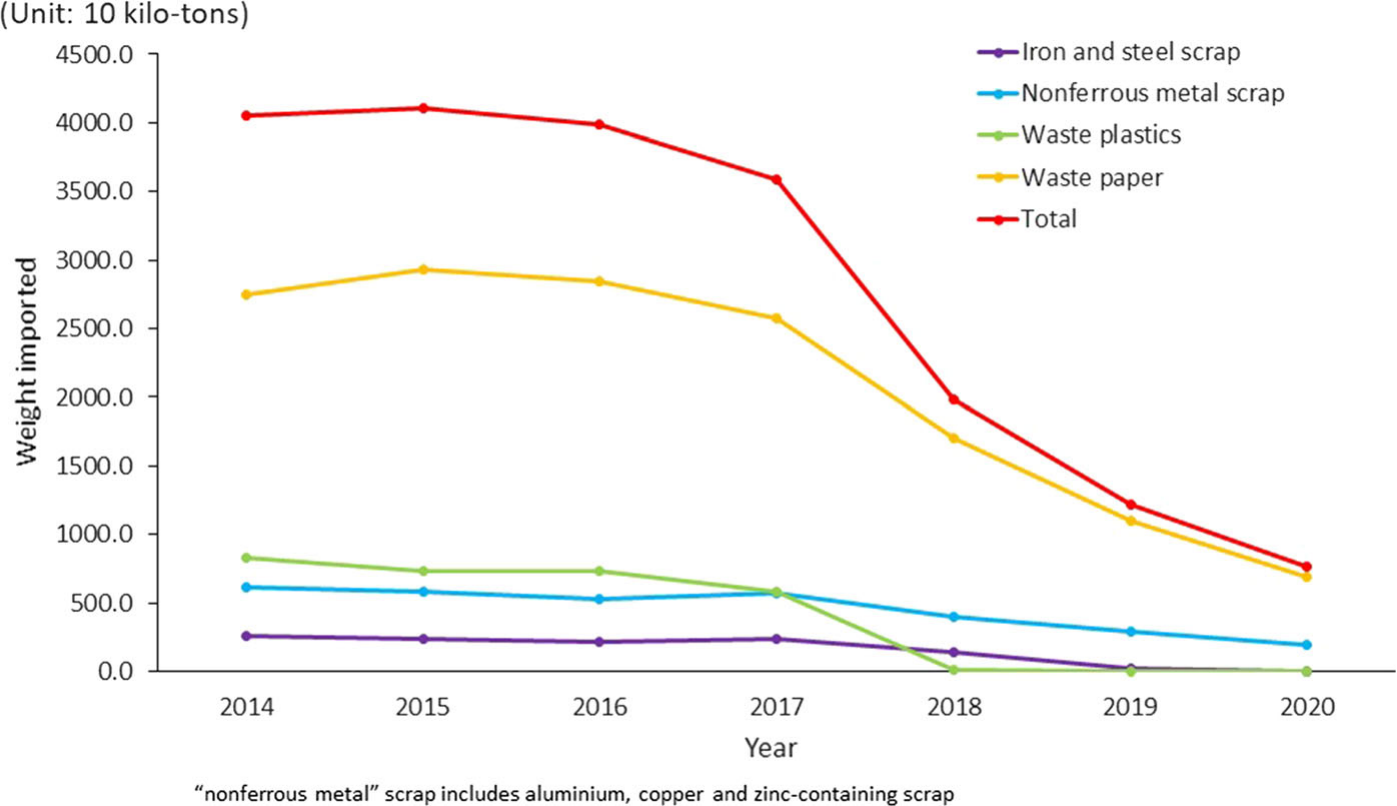
Import data for the main recycled resources to the PRC for the period 2014–2020 (CMC, 2016–2019, Intracen, 2021)

**Fig. 3:**
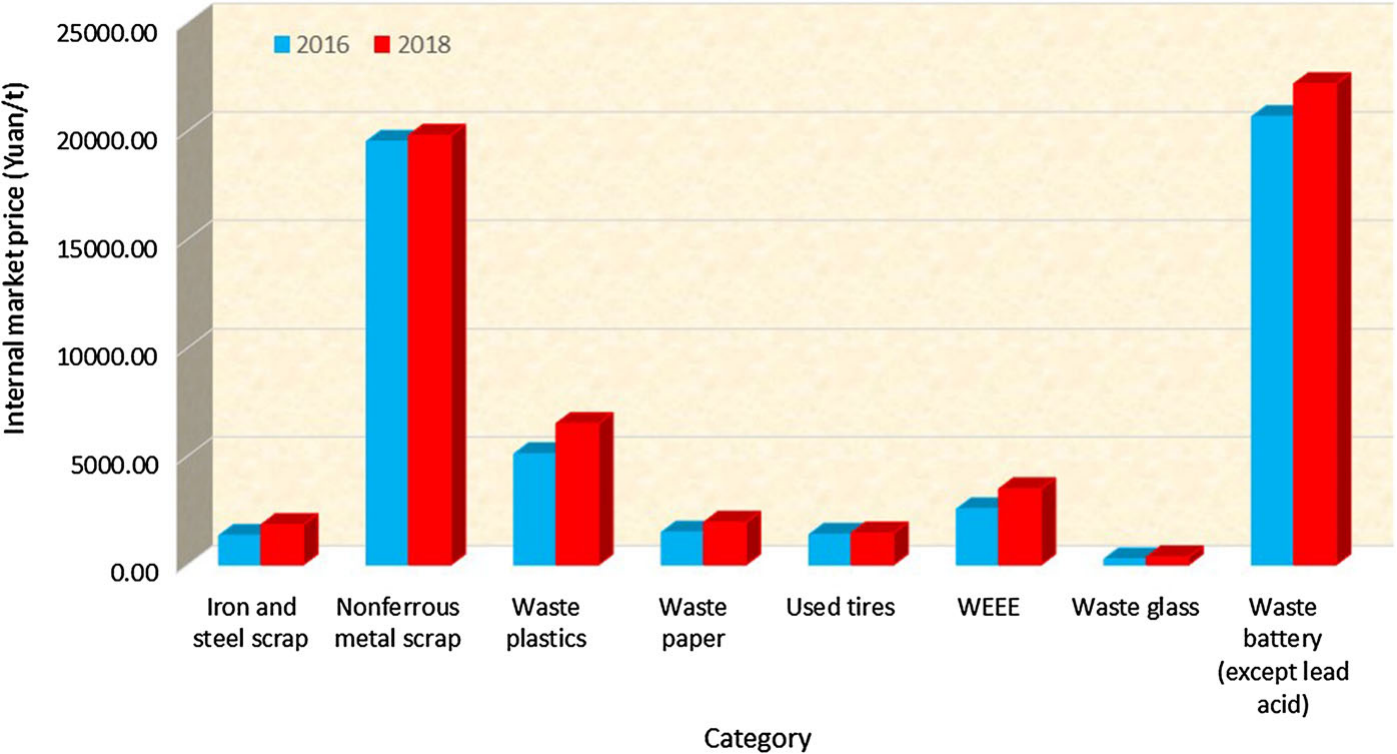
Internal prices of main categories of recyclable waste generated in PRC before and after the ban (unit: Yuan/t)

The review was performed using the following database or website: literature, official report and regulations, etc. in English on Google, Google Scholar, Web of Science; or in Chinese on www.baidu.com, www.cnki.net, www.cqvip.com, www.wanfangdata.com.cn. The data of the resale prices of waste paper for Chinese stakeholders between 2015 and 2018 is collected from references and is presented in Fig. 1. The data in Fig. 2 for the import of renewable resources to the PRC for 2014–2020 is from the China’s renewable resource recycling industry development reports 2016–2019’ (CMC, 2016, 2017, 2018, 2019) and Intracen, 2021. Data in Tables 1 and 2 is also from* China’s renewable resource recycling industry development reports 2016–2019*’ (CMC, 2016, 2017, 2018, 2019). This is presented graphically in Fig. 3.

**Table 1:** The number of main categories of recyclable waste recovered in PRC between 2014 and 2018 (unit: × 10^6^tons) (CMC, 2016, 2017, 2018, 2019)

No.	Waste type	2014	2015	2016	2017	2018
1	Iron and steel scrap^a^	152.30	143.80	151.30	173.91	212.77
	*a. Large iron and steel enterprises*	*88.30*	*83.30*	*90.10*	*147.91*	*187.77*
	*b. Other industries*	*64.00*	*60.50*	*61.20*	*26.00*	*25.00*
2	Non-ferrous metal scrap^b^	7.98	8.76	9.37	10.65	11.10
3	Waste plastics	20.00	18.00	18.78	16.93	18.30
4	Waste paper	44.19	48.32	49.63	52.85	49.64
5	Used tires	4.30	5.02	5.05	5.07	5.12
	*a. Remoulding*	*0.50*	*0.29*	*0.29*	*0.27*	*0.27*
	*b. Recycling*	*3.80*	*4.73*	*4.76*	*4.80*	*4.85*
6	WEEE	3.14	3.48	3.66	3.74	3.80
7	Waste glass	8.55	8.50	8.60	10.70	10.40
8	Waste battery (except lead acid)	0.10	0.10	0.12	0.18	0.19
9	Total	240.55	235.98	246.51	274.02	311.32

^a^The amount of the iron and steel scrap recovered by small and medium iron and steel enterprises and the amount of scrap steel used in the foundry and forging industries have been included in data since 2014

^b^The amount of waste zinc recovered from hot galvanised slag, zinc ash, flue ash, gas mud ash has been included in the statistical scope since 2014

**Table 2:** Main categories of recyclable waste generated in PRC and value of recovered material between 2014 and 2018 (unit: billion Yuan) (CMC, 2016, 2017, 2018, 2019)

No.	Waste type	2014	2015	2016	2017	2018
1	Iron and steel scrap	312.22	198.44	204.26	304.34	392.54
2	Non-ferrous metal scraps	132.47	139.56	182.9	207.9	219.78
3	Waste plastics	110	81	95.78	108.13	118.95
4	Waste paper	61.6	64.27	74.45	97.77	97.02
5	Used tires	6.88	6.51	7.05	7.35	7.48
6	WEEE	7.84	7.83	9.44	12.51	13.3
7	Waste glass	2.57	2.13	2.24	3.21	3.64
8	Waste battery (except lead acid)	1.98	1.85	2.48	3.73	4.21
9	Total	635.56	501.59	578.6	744.94	856.92

**Fig. 4:**
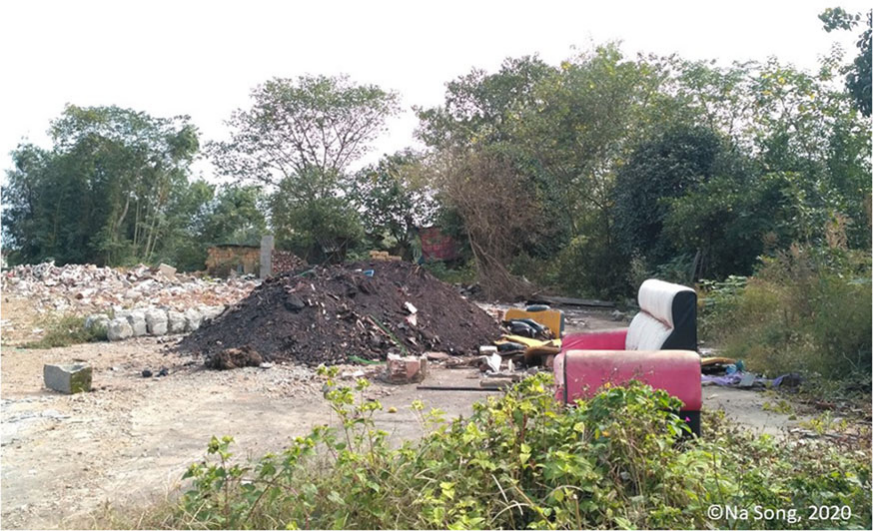
Evidence of increased illegal dumping of domestic and Construction and Demolition Wastes during COVID-19 (photo credit Na Song 2020)

**Fig. 5:**
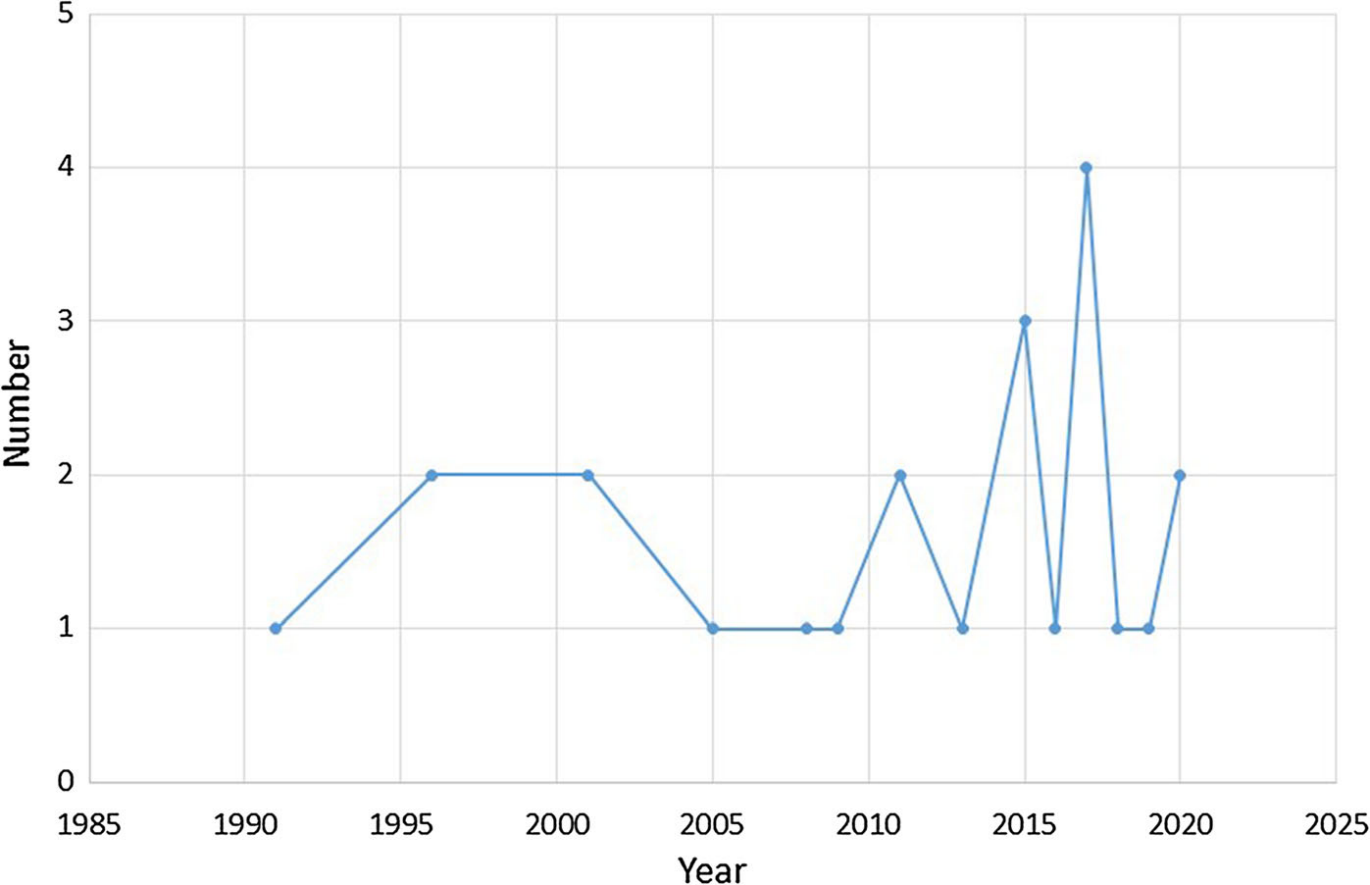
Frequency of revision of national laws and regulations in the PRC (1991–2020)

**Fig. 6:**
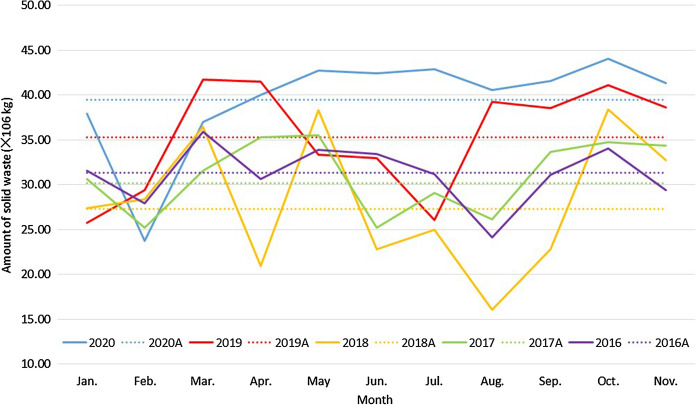
Time series analysis of the amount of solid waste produced for incineration in a southern city of the PRC (2016–2020)

The image in Fig. 4 is a picture taken in 2020 in the countryside of Hetang District, Zhuzhou city, during COVID19. The introduction or revision updates of all policies and regulations on waste management since 1995 have been reviewed in national government databases, and the frequency over time is plotted in Fig. 5. As a case study, the amount of solid waste produced in a southern city in China was recorded for the main disposal route (an incineration plant), and their time series analysis, a potential proxy for wider waste generation is shown in Fig. 6.

## Results and discussion

### Changes in waste import in the run-up to the ban

As shown in Fig. 2, the import of iron and steel scrap and non-ferrous metal scrap both show a consistent decrease from 2014 to 2016, with a slight increase from 2016 to 2017, but then a significant decrease of 42.2% and 30.5%, respectively in 2018. The amount of iron and steel scrap imported was 18.4 × 10 kilo-tons, 2.7 × 10 kilo-tons in 2019 and 2020, respectively (Intracen, 2021), with a decrease of 86.3% and 85.3% compared to the previous year. Whilst the amount of non-ferrous metal scrap imported was 294.9 × 10 kilo-tons and 198.6 × 10 kilo-tons in 2019 and 2020, respectively, with the decrease of 26.2% and 32.7% compared to the previous year. The waste plastic shows a relatively gentle decrease of over 30% from 2014 to 2017, with a reduction in import of more than 99% by 2018 (583 × 10 kilo-tons to 5.0 × 10 kilo-tons), then almost zero in 2019 and 2020. The waste paper imports increase slightly from 2014 to 2015, then decline slowly from 2015 to 2017 with more than 33.8% reduction in 2018, then decline again from 2018 to 2020 with reduction of 35.7% and 37.05% compared with those in the previous year. For the import of iron and steel scrap, non-ferrous metal scrap and waste paper, there are still permitted licences application for companies to import higher levels than standard during the following two years until 1st January 2021 when the Ministry of Ecology and Environment of the People’s Republic of China canceled licenced permission acceptance and permission of permitted licence application (MEE, 2018, 2020).

It should be noted that the ban on imports was not introduced suddenly, rather increasingly strict pollution prevention measures have been implemented since 1996 (Sun, 2019). The import of waste plastic reveals the biggest reduction from 2014 to 2020 with a complete import ban on 1^st^ January 2021 according to (MEE, 2020). Besides, in order to control plastic pollution, an ambitious five-year plan was released on 18th January 2020 to ban or restrict the production, sales, and use of environmentally unfriendly plastic at the Chinese national level (State Council, 2020) which means that regular plastic demand will be cut by 2025 across the whole country. The amount of iron and steel scrap and waste paper imported were categorised in ‘Catalogue of Restricted Import Solid Wastes that Can Be Used as Raw Materials’ (MEE, 2019) and as no licences are being issued in 2021, these products are currently banned. However, it should be noted that import licences may be permitted in the future. The import of non-ferrous metal scrap, higher standard copper-containing waste, and aluminium-containing waste continues at the time of writing (July 2021).

### Reasons why the ban started

One of the main reasons for the ban on waste import is the serious environmental contamination and the associated human health issues derived from handling Waste Electrical and Electronic Equipment (WEEE) imports. For example, prior to the ban, 70% of the WEEE worldwide was imported by China through various paths. Guiyu Guangdong on the southeast coast of China was the biggest site to deal with electrical waste in China, with over a 20-year history of electrical waste recycling. There are fewer than 200 companies approved for e-waste recycling, which means it is impossible to deal with a huge volume of waste in the market. Greenpeace has conducted environmental surveys in Guiyu and surrounding villages since 2005. The test results of these samples consistently show that soil barium (Ba) concentrations in the villages are 20 times that of the background; chromium (Cr) and lead (Pb) content exceeded that of the Chinese standard (Environmental quality standard for soils (GB15618-1995) by > 1,800 times and > 2,000 times, respectively (Xu, 2018).

The mean values of blood lead levels of children in Guiyu were 144.3 ± 69.3 µgL^−1^, whilst 69.9% of children were considered of high lead burden with values of their blood lead levels exceeding 100 µgL^−1^ (Liu, 2009). In addition, organic pollutants such as Polybrominated Diphenyl Ethers) are also widely distributed in Guiyu (Leung et al., 2007) (Li et al., 2019). Local water is so contaminated that residents could only rely on tap water from other places or bottled water. Another reason is that a large amount of unreported WEEE may be illegally shipped to developing countries like China where valuable component materials are recycled inappropriately, impacting directly on human health and the environment (Geeraerts et al, 2015).

One more reason for increasingly stringent regulation is the increasing amount of municipal waste from daily domestic life generated for recycling. The waste arising (mass) of materials commonly recycled for the period 2014–2018 is shown in Table 1 with data presented by value in the domestic market in Table 2 below.

From Table 1, it is apparent that whilst the internal recyclable waste is still substantial, the reduction in imported materials has impacted on waste paper associated with industrial activity, indicating a shortage from 2018. Iron and steel scrap showed an increase from 2015 to 2018, with the similar trend for iron and steel scrap of large iron and steel enterprises whilst the iron and steel from other industries indicated a decrease in the supply chain since 2017.

Finally, the economic development is another reason to promote the implementation of the ban. For example, the import of plastic wastes is a method to mitigate the shortage of resources in China with the plastic wastes being utilised as secondary material from the mid-1990s to the early 2010s when economic development was increased and raw materials demand was rising thanks to Chinese opening-up policy (Shi et al., 2021). Enterprises that engaged in solid waste recycling had been offered tax refunds since 1995 by the Chinese government for economic reasons (Shi et al., 2021). However, with the development of the Chinese economy, the governmental budget to deal with land, water, and air pollution increased; the pressure and costs of environmental remediation have continually increased for the Chinese Government and have exceeded the benefit of waste importation, therefore resulting in the promulgation of the import ban in 2017.

There are only a few companies that get involved in the domestic waste recycling market due to the low revenue streams from domestic recycling, the high cost of wages, the chaotic situation of the domestic waste management system, and the challenge of cheaper ‘secondary resource’ material from waste import. As shown in Fig. 3, prices of main categories of recyclable waste generated in PRC all increased from 2016 to 2018 that might stimulate improvement of the domestic recycling. The import ban is a strategy partially to encourage more people to get themselves involved with domestic recycling and partially to invest more research and development money within waste recycling industries.

### The status of landfill mining in China

Many formal landfill sites were already full in advance of the introduction of new waste import laws. This created significant pressure on the waste management sector for the Chinese Government. A catastrophic landslide event occurred in Shenzhen city, China in 2015 because of overfilling of the landfill, injured 17 people and killed at least 73 people with an estimated total economic loss of more than 0.13 billion USD (Yang et al., 2017). To solve the problems of high cost and limited land to develop a new landfill, landfill mining provides an opportunity to relieve the pressure (Zhou et al., 2015). The import ban aims to reduce the amount of solid waste entering the country, therefore, decreasing the quantity of waste going to landfills, enhancing the environmental footprint and protecting human health. However, landfill mining brings new challenges as the nature of the waste within old landfill sites may be problematic to handle. For instance, excavated plastic bags have impurities even after normal cleaning techniques, this will hinder effective recycling of aged plastic wastes or their use within energy from waste processes such as hydrogenation, gasification, and pyrolysis (Zhou et al., 2014). A further threat may be the most practical processing method of landfill mining plastic wastes is incineration or being treated as residue-derived fuels for energy recovery (Zhou et al., 2014). Moreover, Construction and Demolition Waste (CDW) accounts for 5% of material besides soils (87%) and plastics (2.1%) in the excavated landfills (Hölzle, 2019), which also shows potential problems because of energy consumption as well as emissions resulting from operations, such as excavation, processing and transportation in landfill mining. A report in 2013 shows that about 1 billion tonnes of CDW are generated in China annually, which was five times the amount of municipal solid waste produced in China the same year; the reused and recycled CDW was only 5% (Duan & Li, 2016). Landfill space is reducing rapidly and emissions from previously disposed of wastes increase (Lee et al, 2020), which might lead to higher cost of landfill and more illegal dumping of domestic CDW shown in Fig. 4.

Though the negative impact from landfill mining might have impeded the original aim of the waste ban, the practise of landfill mining would be a choice to reduce landfill space demands, especially for those in big cities where urbanisation expands fast. Moreover, landfill mining could bring more opportunities if technological advances in waste re-processing were accelerated.

Table 1: Main categories of recyclable waste generated in PRC and the amount recovered between 2014 and 2018 (unit: × 10^6^tons).

Table 2: Main categories of recyclable waste generated in PRC and value of recovered material between 2014 and 2018 (unit: billion Yuan).

### The dynamics of domestic policies in China

There are frequent updates of major policies related to waste and the environment since 1991, well before the waste import ban was announced in 2017. As shown in Fig. 3, the frequency of updates or revisions of major policies shows higher level of activity from 2015 onwards. It includes major policy changes related to environmental issues and reflects broader changes in formal environmental regulation intensity (Zhang et al., 2021), which shows significant cross-regional variation in regulation of emission to air, water, and re-use of wastes. The disposal of waste is still dominated by landfills although combustion has increased, and more regulations and policies are being adopted to encourage a more circular economy. The ‘Green Fence’ policy has been implemented since 2013 to restrict copper scrap imports to mainland China and the impact of the policy on the Circular Economy has been assessed and shows that China still has to pay more attention to the domestic recycling industry and keep the import of high-quality copper scrap, which could provide China a transition to a more circular economy on copper in future (Dong et al., 2020). To reduce the source of waste, an announcement on matters related to the ban on the import of solid waste totally (MEE, 2020) was issued on 24th November 2020 by the Ministry of Ecology and Environment (Gov.CN, 2020). It has been implemented since 1st January 2021.

There is increased attention on the transition from fossil fuels to renewable energy sources that not only reduce China’s carbon footprint reduction but also leads to reduction of waste generation and whilst maximising recycling of various waste as secondary carbon raw material, which is the pilot study to develop zero waste cities (GOSC, 2018). There are “11 + 5” cities and regions labelled as zero waste construction pilot city or region alltogether in 2018 on basis of Chinese administrative division, including eleven cities: Shenzhen city, Xining city and so on; one new district: state-level Xiong’an new area in Hebei province; one development zone: Beijing Economic and Technological Development Area; one international cooperative: Sino-Singapore Tianjin Eco-City, one county: Guangze county of Fujian province; one county-level city: Ruijin county-level city of Jiangxi province (Sohu, 2019). Xining city is the only city located in northwest China and the biggest area among all pilot cities. The accumulative budgeted investment for 26 "non-waste cities" construction projects in Xining city is nearly 0.646 billion US dollar. There are 10 solid waste utilization and disposal chains formed within Xining City. The structure of traditional industries such as the chemical industry and refining is optimised to promote industrial recycling, resource utilization, and ecological development, with a reduction of 7.5% on energy consumption per unit of GDP compared to last year.

In terms of agriculture, five production bases are built in the implementation of the agricultural and livestock product quality and safety assurance project and it occupied 57,730 ha, including the national green food raw material (broad bean) standardised production base for broad bean and potato in Huangzhong District and Datong County, respectively. Through using alternative technologies such as using organic fertilizers, crop rotation, and improved irrigation, there has been a reduction of 43,000 ha of land that has been treated with chemical fertilizers and pesticides. In this model, 630,000 acres of chemical fertilizers and pesticides have been reduced, and 77,000 tons of organic fertilizers have been subsidized to increase efficiency in 2020. The use of chemical fertilizers and pesticides in 2020 was reduced by 41.9% and 32.9%, respectively compared with those of 2018. In addition, "Enterprise recycling, farmer participation, government supervision, and market promotion" agricultural film recycling system is also built-in Xining city with the recycling rate of the agricultural film more than 90%. For citizens daily life, the domestic waste classification in Xining city covers a total of 304,000 households with a recycling rate of 37% on domestic waste, including more than 1,300 tons of waste in plastic, textile, metal, paper, electronic products, and other recyclables and perishable waste that enter the recyclable and non-hazardous disposal network separately.

Land use policy is further impacted by recent plans to develop zero waste cities (Lee et al., 2020): a large amount of land will be used more efficiently due to less industrial solid waste exposure. Through the establishment of green waste-free cells in society, promotion and guidance of the concept of waste-free are vigorously promoted to form a consensus of waste-free for citizens. According to the investigation conducted by the Qinghai Provincial Social Situation and Civil Investigation Center, the popularisation rate of publicity, education, and training for the construction of a "waste-free city" in Xining City is 85.34%. The degree of satisfaction for the construction of the "waste-free city" was 83.02% for the public. The construction of green cells consists of green posts, green restaurants, green mines, and green buildings, etc.

In addition, more regulations and policies have been introduced since 1995 to restrict the waste import properly, which shows that the Chinese government has set a basic regulation frame all the time and is trying to build a better recycling or waste management system over time.

### Challenges and opportunities for waste management companies

The life cycle cost of recycled paper manufacturing in China has been assessed (Li et al., 2020). A ban on unsorted recovered paper was announced in 2018 and the import quota for recovered paper has been tightened, leading to a dramatic drop in the amount of imported recovered paper (Li et al., 2020). As the recovered paper is a key raw material for the recycled papermaking industry, alternatives (e.g., straw, imported wood pulp, and imported deinked pulp) are now being assessed to deal with the decrease in the feedstock. The results indicated that imported deinked pulp might be the trend for the recycled papermaking industry in China with a growth in the price of the domestic recovered paper (Li et al., 2020). This change might bring additional research and development of potential alternatives in the future to satisfy the increasing need for imported recycled material due to the waste import ban.

The environmental impact on the life cycle of used polyethylene terephthalate (PET) was also analysed under many post-ban scenarios (Ren et al., 2020). The result shows that the absence of imported used PET in China leads to an increase in virgin PET fibre production using manufacturing processes that are based on carbon-intensive coal. This brings an additional environmental impact because of the higher air pollution emissions from production (Ren et al., 2020). The treatment of air pollution may become new challenge; however, improving the local PET recycling rates and searching for production alternatives are emerging new opportunities.

There are recycled commodities, which may be sold at a cheaper price to downstream companies because of higher quality compared to domestic products. Without importing waste material, the costs of downstream companies would increase because they cannot use cheaper recycled items directly, or they need to find an alternative source. However, with more initiatives available to boost collection and recycle businesses (Zuo et al., 2020), there are now opportunities for people to start new, or adapt current, businesses in different ways and bring new jobs.

Businesses who deal with waste still need a significant and consistent supply of waste materials, which pushes the domestic waste recycling companies to move their attention to domestic waste as an alternative, for example, domestic copper scrap recycling and application (Dong et al., 2020; Liu et al., 2020; Wang et al., 2019). Some of the larger companies may survive after small businesses fail: the trash ban is a threat to the existence of small companies; however, it can be an opportunity for a big company to enlarge and update its technology (Zhang, 2020a). Some companies will change their processing to higher efficiency and value to survive, for example, developing a new idea to apply the recycled resource. It may even force companies to consider product recycling at the design stage, such as extended producer responsibility (EPR) (Zhang, 2020b). Therefore, instead of just disposing of the waste directly and paying fees to get the raw recycled waste material directly from abroad, companies are pushed to design products, which consider new waste sorting and recycling methods.

For waste management companies in Southeast Asian countries, an increase in solid waste import after the China import ban is now a threat to business sustainability from the potential imposition of similar restrictions by their governments. There is no detailed data covering company failure as a result of the ban and the number of jobs lost, which is quite important for the national-society. However, it seems that some Southeast Asian countries have already tightened their restrictions by announcing statements or revoking their import licences for local companies that process plastic waste or e-waste (WMR, 2018).

For companies in mainland China, technologies such as the Beidou navigation system, the Internet of Things, Internet and Artificial Intelligence, etc. are applied by Yixun Intelligent Environmental Sanitation System to manage the whole process of people, trucks, materials, machines involved in environmental sanitation management in real-time. It can help design environmental sanitation management models, rationally, improve the quality of sanitation operations, reducing environmental operation costs, and assessing the effectiveness of the management through data (Gov.CN, 2019). It is a new start and possibility for the structure of the domestic waste management system thanks to the involvement of the Internet and big data. Whilst some information has been derived from structural changes of the global waste trade network (Wang et al., 2020) (Pu et al., 2019; Tran et al., 2021), and mechanisms of response (Tan et al., 2018), ‘Big data’ application for the waste management systems could be a potential bright path for the transition of domestic companies.

### Impact of the COVID19 pandemic on waste management system

The global pandemic has influenced the production and management of the various industrial sectors, in terms of waste to recycling generation of end-of-life vehicles were stopped because of the shutdown of companies upstream, and there was an insufficient supply of raw materials for many manufacturing companies. Whilst for domestic WEEE, there is a consequential increase in the generation of e-waste due to increasing utilization of electrical and electronic devices for online teaching or work from home during the pandemic or post COVID19 in a developing country (Adejumo & Oluduro, 2021). In terms of waste to energy, for example, waste incineration for power generation (business), the closure of restaurants and service companies leads to the amount of food collection and volume of transport in some cities or regions was only 25% ~ 33% of pre-pandemic levels of pre-pandemic levels due to lockdown and social restrictions. On the other hand, the pandemic caused an increase in medical waste due to increased production of protective materials (masks, gowns, etc.). The more complicated situation poses a potential challenge for waste management system because of shortage of the raw materials for manufacture, restricted traffic, infection risk, higher cost. In addition, large-scale sanitation and disinfection work during the pandemic increased company operating costs such as labour, machinery, materials, and transportation (Finance, 2020). However, studies have shown that lower levels of industrial wastes are generated because of lower production and exports due to reduced demand during the shutdown (Maliszewska et al., 2020). More waste has been sent directly to incineration in order to reduce the risk of infection, resulting in the over-working of incineration equipment in some cities and regions. Besides, there is also the impact of the extended construction period, increased investment, and secondary urban environmental protection issues on projects under construction of waste incineration for power generation. According to the current local control levels, the construction period has been impacted with an average of 55 days. The overlap of the processing period is longer because of the lag in the impact of materials, equipment, logistics, and other parts of the supply chains. In addition, the investment will also increase due to the following: (i) the increase in the cost of personnel, machinery, and materials of the project, (ii) the increase in the cost of capital, and (iii) the loss of operating income caused by the delay in commissioning (Finance, 2020). Higher costs and lower-income bring a dilemma for investors and the construction of waste incineration facilities in the future. It is difficult to make decisions on the treatment of the municipal solid waste and medical waste, to consider separate or not during the difficult periods during the pandemic.

The total mass of the portion of municipal solid waste from a city in Southern China, transported to a waste to energy incineration plant is shown in Fig. 6. The annual average and detailed monthly amounts received between 2016 and 2020 are presented. As a yearly average, a slight increase in 2019 from roughly comparable levels in 2016–2018 is enhanced in 2020. However, the detailed monthly variation is also informative. In general, there is a big drop in the amount of solid waste produced in February because of the lockdown (coming into effect early in 2020 in China) and people returning to their hometown for the Spring Festival. In pre-pandemic, variation across the year tends to show decrease in amounts over the popular summer holiday months, picking up particularly in the period of the early autumn festivals. For 2020 this variation is suppressed and a steady increase in waste generation is seen as restrictions on movement, maintain a constant population in the city and consumption rates of produce increase along with increased disposal of PPE. Changes in annual average waste production prior to the pandemic are less easy to rationalise, and the dip from 2016 to 2018 and increase from 2018 to 2020 may be partly attributed to the waste import ban and further restrictions.

The pandemic increases pressure on the waste management system on top of the waste ban and implementation of a series of related regulations and policies. However, it also provides some dynamic evidence of the response of the waste management system. The post-pandemic recovery should provide an opportunity to establish more resilient, sustainable management systems in the future.

## Conclusion and recommendations

The impact of the waste ban and new policies on the waste management sector in mainland China has been reviewed in the context of recent challenges from the COVID19 pandemic as national legislation prevented the import of waste. We highlight the landfill mining situation in China, assessing the dynamics of domestic policies before and after the implementation of the ban in China and challenges and opportunities for waste management companies. The integrated assessment of the impact of the ban in China has relevance for future influence on the waste management system in China.

In conclusion, the change in waste import in the run-up to the ban leads to different situations for the variety of waste imports in the future in China. The ban drove price increases in the domestic recycling sector that forces companies to introduce more formal recycling processes and to drive the consumption behaviours to more reasonable and environmentally friendly options. The impact in China is ultimately to reduce the pollution of the environment and improve health, but the negative impact from landfill mining has impeded the original aim of the waste ban that requires further technological development. The dynamic of domestic policies in China shows a higher level of activity of updates and revisions or promulgations of new policies from 2015 onwards and ‘zero waste cities’ brings new hope for improvement of the Chinese waste management system. Lessons could be learned for other countries, such as countries in Southeast Asia, after the import ban in China through discussion on challenges and opportunities for waste management companies in China. ‘Big data’ application could be a transition for the domestic companies. Whilst the restrictions on imports have provided a stimulus for improved waste management practices, changes in those restrictions in other territories may have further impacts on the economic viability of businesses once these countries lift the restrictions similar to the ban promulgated in China. Moreover, the pandemic brings more tricky challenges for the waste management system than before; however, it also gives a clue to the world that how quickly the waste management system can respond, particularly post-pandemic will be an important step to establishing sustainable management systems in the future.

The detailed analyses of the companies that ceased trading or of the rise in unemployment in the sector due to the waste ban, the consideration of advantages and disadvantages on ‘zero waste city’ and ‘big data’ application would be urgent priorities for further research.

## Data Availability

Data used in this paper was derived from publicly available information and the result of ongoing research. No personal or sensitive information was used.
